# Excessive Intake of High-Fructose Corn Syrup Drinks Induces Impaired Glucose Tolerance

**DOI:** 10.3390/biomedicines9050541

**Published:** 2021-05-12

**Authors:** Hidemi Hattori, Yuma Hanai, Yuto Oshima, Hiroaki Kataoka, Nozomu Eto

**Affiliations:** 1 Department of Biochemistry and Applied Biosciences, Faculty of Agriculture, University of Miyazaki, 1-1 Gakuenkibanadai-nishi, Miyazaki 889-2192, Japan; yumhanai@gmail.com (Y.H.); gc17006@student.miyazaki-u.ac.jp (Y.O.); neto@cc.miyazaki-u.ac.jp (N.E.); 2 Section of Oncopathology and Regenerative Biology, Department of Pathology, Faculty of Medicine, University of Miyazaki, Miyazaki 889-1692, Japan; mejina@med.miyazaki-u.ac.jp

**Keywords:** impaired glucose tolerance, nonobesity, high-fructose corn syrup drinks, insulin

## Abstract

The number of patients with diabetes was approximately 463 million worldwide in 2019, with almost 57.6% of this population concentrated in Asia. Asians often develop type 2 diabetes (T2D), even if they are underweight and consume a smaller amount of food. Soft drinks contain large amounts of sweeteners, such as high-fructose corn syrup (HFCS). Excessive intake of HFCS drinks is considered to be one of the causes of T2D. In the present study, we investigated the effect of excessive consumption of HFCS–water on glucose tolerance and obesity under conditions of controlled caloric intake using a mouse model. Three-week-old male ICR mice were divided into two groups and given free access to 10% HFCS–water or deionized water. The caloric intake was adjusted to be the same in both groups using a standard rodent diet. The excess HFCS–water intake did not lead to obesity, but led to impaired glucose tolerance (IGT) due to insulin-secretion defect. It affected glucose and fructose metabolism; for example, it decreased the expression of glucokinases, ketohexokinase, and glucose transporter 2 in the pancreas. These results suggest that excessive consumption of HFCS drinks, such as soft drinks, without a proper diet, induces nonobese IGT due to insulin-secretion defect.

## 1. Introduction

The number of patients with diabetes worldwide was estimated to be approximately 463 million in 2019, and is expected to reach 700 million by 2045 if appropriate control measures are not taken [1,2]. More than 90% of all patients with diabetes have type 2 diabetes (T2D), which results from a complex inheritance–environment interaction along with other factors, such as obesity and lifestyle. Almost 57.6% of the population with diabetes is concentrated in Asia [2], with one of the reasons for this observation being the genetic makeup of the population in this region. Genomewide association studies have dissected the genetic architecture of T2D, and more than 400 genetic variants of genes, such as *UBE2E2, ZFAND3,* and *KCNQ1*, have been implicated in the risk of T2D [3]. However, not all cases of T2D are related to these genes. Obesity is one of the causes of T2D. However, it is pertinent to mention that the obesity rate in Asia is low compared to the international standards [4,5,6]. In fact, Japanese patients with T2D have an average body mass index (BMI) of 23.1, which is lower than the BMI (29.4) of patients in Western countries [7]. Therefore, obesity is not necessarily related to the development of T2D. The development of T2D is attributed to impaired insulin secretion, insulin resistance (IR), or both. Among Japanese patients with T2D, the prevalence of impaired insulin secretion is considerably higher than that of IR [8]. Because of the difference in the pathogenesis of T2D, we believe that preventive measures need to be tailored to each case. Moreover, normal-weight patients with metabolic disorders, referred to as “metabolically unhealthy normal weight” (MUNW), have also become a cause of concern. It has been reported that MUNW patients in Korea are at a higher risk of developing T2D than metabolically healthy obese individuals [9]. Therefore, a normal body weight might not be reflective of the actual health status of an individual.

For in vivo studies on T2D, obese mice with disrupted satiety signals, such as *db/db* and *ob/ob* mice, or obese T2D mice fed a high-fat diet containing fat amounts unachievable through a normal diet, have often been used. Because T2D develops even with low energy intake, it is suggested that the disease is caused by factors other than obesity or overeating. The average daily energy intake varies by country; for example, the average daily energy intake in Brazil is 2855 kcal [10], whereas in Japan it is 1903 kcal [11]. It is therefore pertinent to focus on energy intake in studies on T2D.

Excessive intake of sweetened soft drinks has been reported to be associated with a relatively higher risk of T2D and obesity [12,13,14]. Sweetened soft drinks contain large amounts of sweeteners, such as high-fructose corn syrup (HFCS). HFCS was developed in the 1960s as a liquid sweetener to replace sucrose, and it was introduced to the food and beverage industry in the 1970s. The use of HFCS in soft drinks has increased rapidly owing to its inexpensive and sweet and tasty properties. The intake of HFCS-containing sweetened soft drinks induces gain in body weight and adipose tissue hypertrophy [15,16], and increases fasting blood glucose [17] and blood triglyceride levels [18]. On the contrary, there are reports that fructose-containing sugar intake is not associated with the development of T2D and obesity [19,20]. There is thus a disagreement on whether HFCS can be a direct risk factor for T2D or obesity. However, the caloric intake has been considered in only a few of these studies. Furthermore, few studies have been conducted to evaluate the effects of excessive intake of HFCS drinks on T2D and obesity under the same conditions of caloric intake. Therefore, it is unclear whether HFCS directly induces diabetes or whether excessive energy intake causes obese T2D.

With the objective of clarifying the relationship between excessive consumption of HFCS-containing soft drinks and T2D, in the present study, we investigated the effect of excessive consumption of HFCS–water on glucose tolerance and obesity under conditions of controlled caloric intake.

## 2. Materials and Methods

### 2.1. Animals

This study was approved by the ethics committee of Miyazaki University (Miyazaki, Japan; No.2019-031-2, approved 17 December 2019), and the protocol was consistent with the committee’s guidelines for the care of animals. Three-week-old male ICR mice were purchased from SLC Japan, Inc. (Shizuoka, Japan) and individually placed in plastic cages under controlled atmospheric conditions (12 h light/12 h dark (07:00–19:00), 20–22 °C). HFCS-55 (Nihon Shokuhin Kako Co., Ltd., Tokyo, Japan) including 55% fructose was used. The mice were randomly divided into two groups (*n* = 6) and were given free access to 10% HFCS–water (HFCS group) or deionized water (control group). The amount of HFCS–water intake was measured daily, and the caloric intake was adjusted to be the same for 8 weeks, using a standard rodent diet (Rodent LabDiet EQ 5L37, Japan SLC, Inc., 3.38 kcal/g). Body weight was measured weekly. For measurement of BMI, body length was measured from the nose to the anus.

### 2.2. Oral Glucose and Fructose Tolerance Tests (OGTT and OFTT) and Insulin Tolerance Test (ITT)

For OGTT and OFTT, mice were fasted for 6 h, and then blood glucose was measured via a cut in the tail using a glucose meter (Glucose PILOT, Iwai Chemical Co., Tokyo, Japan). Thereafter, 2.0 g of glucose or fructose per kg body weight was orally administered immediately, and blood glucose was measured at 15, 30, 60, and 120 min after the oral administration. For measurement of blood insulin levels, the blood collected from the tail vein with a heparinized capillary tube was centrifuged (1700× *g*, room temperature, 15 min), and the serum insulin level was measured using a mouse insulin ELISA kit (FUJIFILM Wako Shibayagi Co., Gunma, Japan). For ITT, mice were intraperitoneally injected with 1.0 U/kg body weight of insulin (Humulin R; Eli Lilly Japan K.K., Hyogo, Japan) without fasting. Blood glucose levels were measured at 0, 15, 30, 60, and 120 min after the injection from the tail vein. The homeostasis model assessment of insulin resistance index (HOMA-IR) was calculated as fasting blood glucose (mg/dL) × fasting serum insulin (µU/mL)/405.

### 2.3. Measurement of Tissue Weight and Serum Parameters

After 6 h of fasting, 2.0 g/kg glucose was administered orally. The mice were anesthetized with 2% isoflurane (Abbott Japan LLC., Tokyo, Japan) mixed with air and oxygen using a vaporizer, the heart was exposed, and the cardiac blood was collected 30 min after the oral administration. Thereafter, the liver, pancreas, and epididymal and subcutaneous fat were removed, and their weights were measured. The collected blood was centrifuged (1700× *g*, room temperature, 15 min), and the serum was stored at −30 °C until analysis. The levels of serum triglyceride (TG), total cholesterol (T-Cho), aspartate transaminase (AST), alanine transaminase (ALT), blood urea nitrogen (BUN), uric acid (UA), and total protein (TP) were measured using a Fuji DRI-CHEM NX700V (Fujifilm Co., Tokyo, Japan).

### 2.4. RNA Isolation and Quantitative PCR (qPCR)

The pancreas was crushed using Lysing Matrix D (MP Biomedicals Germany GmbH, Eschwege, Germany) and ISOGEN II (Nippon Gene Co., LTD, Tokyo, Japan) at 4 °C, and total RNA was extracted following the instructions provided with ISOGEN II. The cDNA was synthesized from total RNA using ReverTra Ace (Toyobo Inc., Osaka, Japan). The qPCR analysis was carried out using Luna Universal qPCR Master Mix (New England Biolabs, Inc., Ipswich, MA, USA) and primer sets (Table 1) on an Aria Mx Real-time PCR System (Agilent Technologies, Inc., Santa Clara, CA, USA). Target gene expression was normalized to that of 18S rRNA, and the relative expression was calculated using the ΔΔCt method.

### 2.5. Histological and Immunohistochemical Analyses

The liver, pancreas, and epididymal and subcutaneous fat were fixed in 10% (*w/v*) formaldehyde solution for 2 days. All the specimens were embedded in paraffin and cut into 4 μm-thick coronal sections. The sections were stained with hematoxylin and eosin (HE) and with antibodies specific for insulin. They were first washed in phosphate-buffered saline (PBS) at pH 7.2 for 5 min. The sections were then incubated with the anti-insulin mouse monoclonal antibody (Abcam plc, Cambridge, UK) in 10% skim milk overnight at 4 °C. Thereafter, the sections were rinsed in PBS, three times for 5 min each time, and then incubated with goat antirabbit immunoglobulin/horseradish peroxidase (Agilent Technologies) for 30 min at room temperature. After rinsing with PBS three times for 5 min, immune complexes were visualized by the addition of Simple Stain DAB Solution (Nichirei Corp., Tokyo, Japan). After rinsing with distilled water, the nuclei were stained with Carrazzi’s hematoxylin solution (FUJIFILM Wako Pure Chemical Co., Osaka, Japan). The percentage of insulin-positive area was measured using the image-analysis software ImageJ (https://imagej.nih.gov/ij/index.html, accessed on 8 April 2020).

### 2.6. Measurement of Cytokine Levels in the Serum

The levels of serum keratinocyte-derived chemokine (KC), tumor necrosis factor (TNF)-α, monocyte chemoattractant protein (MCP)-1, and adiponectin were measured using commercial kits (R&D Systems, Minneapolis, MN, USA), as described by the manufacturer.

### 2.7. Statistical Analysis

All the data are expressed as mean ± SEM. The R software (version 4.0.3, http://www.r-project.org, accessed 27 October 2020) was used for statistical analysis, and the data were statistically analyzed using Student’s *t*-test or Welch’s *t*-test. A value of *p* < 0.05 was considered significant.

## 3. Results

### 3.1. Excess HFCS–Water Intake and its Nutrient Intake Rate under Conditions of Controlled Caloric Intake

**Figure 1:**
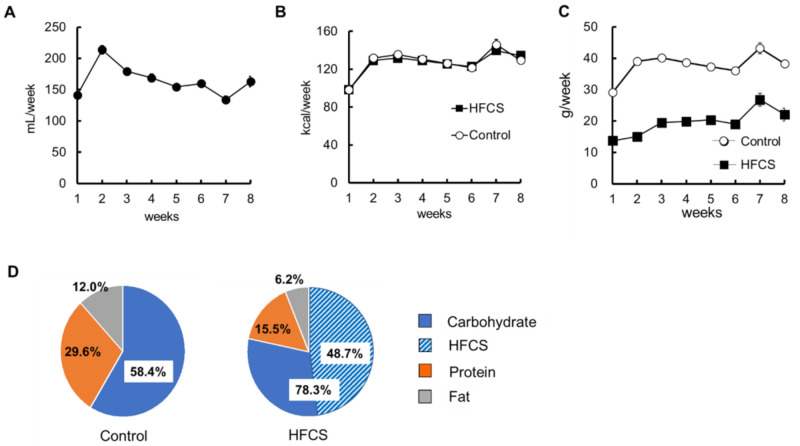
High-fructose corn syrup (HFCS)–water intake, caloric intake, diet intake weight, and calorie rates of nutrient. (**A**) HFCS–water intake per week. (**B**) Energy intake per week. (**C**) Intake weight of 5L37 per week. (**D**) Percentage of intake calories of carbohydrate, protein, and fat ingested for 8 weeks. All the data are expressed as mean ± SEM. *n* = 6.

The amount of HFCS–water intake was measured daily because it varied on a daily basis. Figure 1A shows the amount of HFCS–water intake per week for 8 weeks. A trend of higher intake was observed in the second week after the drink was switched to HFCS; however, the intake was almost constant over the 8 weeks. Mice were pair-fed with the standard rodent diet (5L37). The calorie content of HFCS was 2.76 kcal/g, and mice in the HFCS and control groups were fed 5L37, so that the calories would be the same for 8 weeks (Figure 1B). Figure 1C shows the intake weight of 5L37 per week for 8 weeks. According to the attached document of 5L37, the total calorie content comprises 29.6% protein, 12.0% fat, and 58.4% carbohydrate. The control group consumed the diet at this nutrient ratio. On the other hand, in the HFCS group, HFCS accounted for 48.7 % of the total caloric intake, and the total carbohydrate content was 78.3%, protein 15.5%, and fat 6.2% (Figure 1D). The HFCS group had a high ingestion rate of carbohydrates and a low ingestion rate of protein and fat compared to the control group. Although mice in the HFCS group had an excessive intake of glucose and fructose, the total caloric intake was the same as that in the control group. The nutrient intake in the HFCS group was imbalanced.

### 3.2. Excess HFCS–Water Intake under Conditions of Controlled Caloric Intake Did Not Lead to Obesity

**Figure 2:**
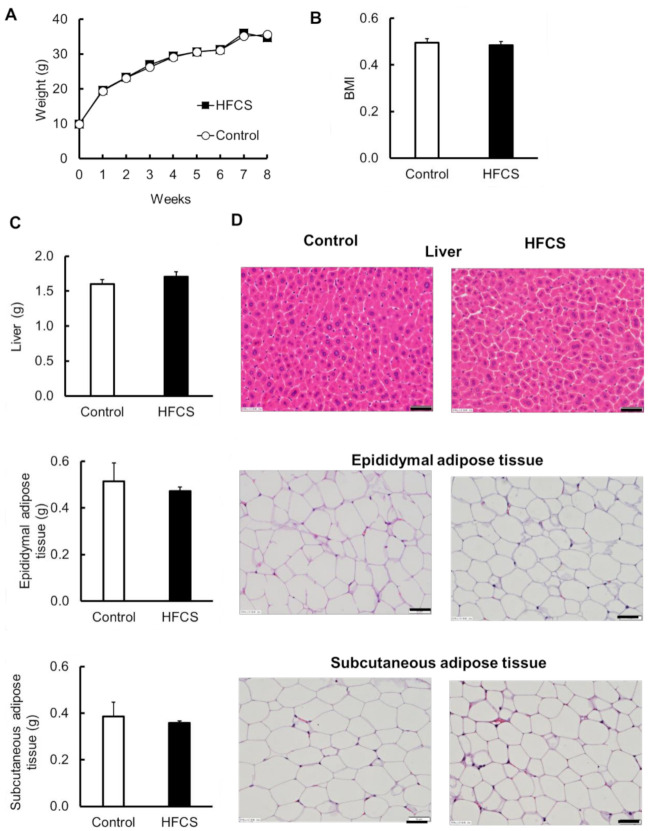
Physical parameters and histological observation of the liver and epididymal and subcutaneous adipose tissues. (**A**) Body weight. (**B**) Body mass index (BMI) at the end of the experiment. (**C**) The weight of liver and epididymal and subcutaneous adipose tissues. (**D**) Hematoxylin and eosin-stained sections of liver and epididymal and subcutaneous adipose tissues. Bar = 50 µm. All data are expressed as mean ± SEM. *n* = 6.

Body weights were measured for 8 weeks (Figure 2A). At the end of the experiment, the body weights of mice in the HFCS and control groups were 34.7 ± 0.44 g and 35.6 ± 1.09 g, respectively, and there was no difference between the groups (Figure 2A). There was also no difference in BMI between the two groups (Figure 2B). In addition, no differences in appearance between the two groups were observed. To investigate the effects on the organs, the weights of the liver and the epididymal and subcutaneous adipose tissues were measured, and histopathological examination of HE-stained sections was performed. The weight of each organ was not significantly different between the two groups (Figure 2C), and lipid accumulation in the liver and differences in the size of adipocytes in the adipose tissues were not observed (Figure 2D). The intake of excess HFCS–water under conditions of controlled caloric intake did not lead to obesity.

### 3.3. Excess HFCS–Water Intake under Conditions of Controlled Caloric Intake Led to Impaired Glucose Tolerance (IGT)

**Figure 3:**
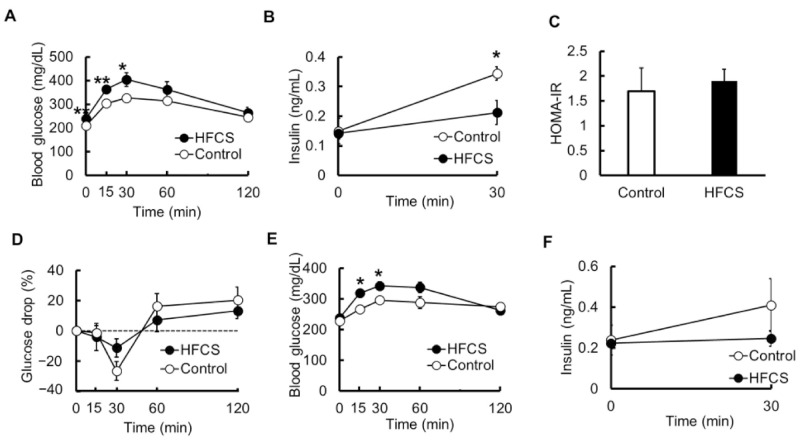
Changes in glucose and insulin levels in the blood. (**A**) The change in the blood glucose levels with time determined using the oral glucose tolerance test (OGTT) (*n* = 6). (**B**) Insulin levels at 0 min (immediately before administration) and 30 min determined using the OGTT (*n* = 5–6). (**C**) Homeostasis model assessment of insulin resistance index (HOMA-IR) was calculated as fasting blood glucose (mg/dL) × fasting serum insulin (µU/mL)/405 (*n* = 6). (**D**) The change in the glucose levels with time determined using the insulin tolerance test (ITT). The data are expressed as percentage of values at 0 min (immediately before administration) (*n* = 4). (**E**) The change in the blood glucose levels with time determined using the oral fructose tolerance test (OFTT) (*n* = 5–6). (**F**) Insulin levels at 0 min (immediately before administration) and 30 min determined using the OFTT (*n* = 4–5). All data are expressed as mean ± SEM. ** *p* < 0.01, * *p* < 0.05 vs. control (Student’s *t*-test).

To investigate glucose tolerance, we performed OGTT. The fasting blood glucose level was higher in the HFCS group than in the control group, and the glucose levels at 15 and 30 min after oral glucose administration were significantly higher (Figure 3A). Next, serum insulin levels were examined at 0 min (immediately before administration) and 30 min after oral glucose administration. Insulin levels during fasting were the same in both groups. However, the level after 30 min increased to 2.31-fold in the control group, but only to 1.50-fold in the HFCS group; insulin secretion under glucose tolerance was significantly lower in the HFCS group than in the control group (Figure 3B). The HOMA-IR calculated from the fasting blood glucose levels and insulin levels did not differ between the two groups (Figure 3C). Moreover, ITT was performed to explore the effects of insulin. When the glucose level was measured over 120 min after insulin injection, the level was not different between the two groups (Figure 3D). Furthermore, we investigated whether the blood glucose level increased even when fructose was ingested. Interestingly, oral fructose administration also increased the blood glucose levels, and the levels after 15 and 30 min were significantly higher in the HFCS group than in the control group (Figure 3E). At fasting and 30 min after the oral fructose administration, the insulin level was not statistically different between the two groups; however, it increased to 1.63-fold in the control group, with almost no change in the HFCS group (Figure 3F). The intake of excess HFCS–water intake under conditions of controlled caloric intake led to IGT not because of IR, but because of insulin-secretion defect.

### 3.4. Serum Biochemical Parameters

To investigate the effects on serum biochemical parameters besides glucose, the levels of TG, T-Cho, UA, BUN, AST, ALT, and TP in the serum were measured (Table 2). TG and UA levels were not different between the two groups. The levels of BUN, ALT, and TP were lower, and AST levels tended to be lower in the HFCS group than in the control group. Although mice in the HFCS group, with poor nutritional balance, had a high ingestion rate of carbohydrates and a low ingestion rate of proteins and lipids compared to those in the control group, and the levels of T-Cho tended to be higher in the HFCS group.

### 3.5. Effects on Pancreas

**Figure 4:**
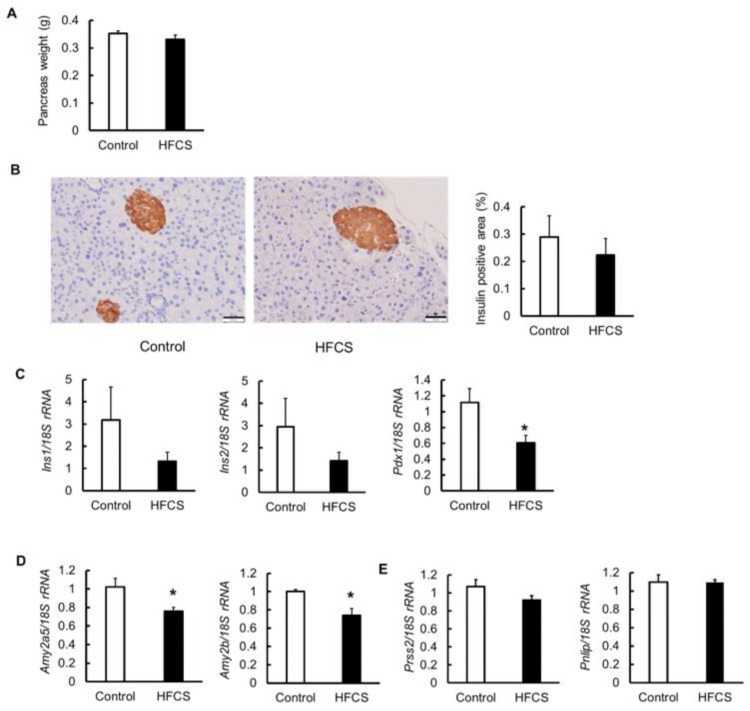
Effects of high-fructose corn syrup (HFCS)–water intake on the pancreas. (**A**) Weight of pancreas. (**B**) Immunostaining of insulin (brown) and the percentage of insulin-positive area (islets of Langerhans) of the pancreas. Bar = 50 µm. (**C**) mRNA expression of insulin 1 and 2 (*Ins1* and *2*), and pancreatic and duodenal homeobox 1 (*Pdx1*). (**D**) mRNA expression of the amylase, *Amy2a5* and *Amy2b*. (**E**) mRNA expression of trypsin 2 (*Prss2*) and lipase (*Pnlip*). All data are expressed as mean ± SEM. *n* = 6. * *p* < 0.05 vs. control (Student’s *t*-test).

Because the secretion of insulin 30 min after the oral glucose administration was lowered in the HFCS group, the effects on the pancreas were investigated. The weight of the pancreas did not differ between the two groups (Figure 4A). The morphological characteristics and the percentage of insulin-positive area (islets of Langerhans) were also not different between the two groups (Figure 4B). Next, the expression of insulin (*Ins*) 1 and 2 and pancreatic and duodenal homeobox 1 (*Pdx1*) mRNAs was measured (Figure 4C). There was no statistical difference, and the expression of *Ins1* and *Ins2* tended to be lower in the HFCS group than in the control group. The expression of *Pdx1*, an insulin promoter factor, was significantly downregulated in the HFCS group. Serum pancreatic amylase levels are known to be decreased in patients with diabetes [21,22]. Therefore, the mRNA expression of amylase, which is also a marker of pancreatitis, was measured. The expression of both *Amy2A5* and *Amy2B* in the pancreas was lower in the HFCS group than in the control group (Figure 4D). In addition to amylase, pancreatic exocrine cells also secrete digestive enzymes, such as trypsin and lipase. Therefore, we measured the expression of trypsin 2 (*Prss2*) and lipase (*Pnlip*); however, there was no difference between the two groups (Figure 4E). The HFCS group had impaired pancreatic function.

### 3.6. MRNA Expression of Glucose Transporter 2 (Glut2), Glycolytic Enzymes, Amino Acid Transporter, and Glutaminolysis in the Pancreas

**Figure 5:**
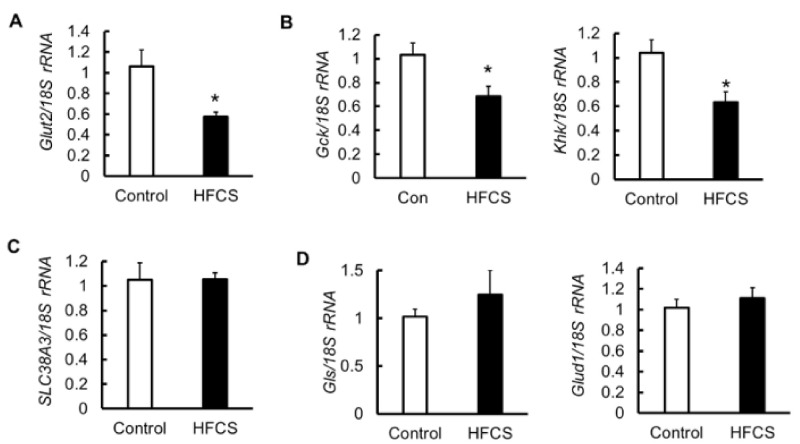
mRNA expression of glucose transporter 2 (*Glut2*), glycolytic enzymes, amino acid transporter, and glutaminolysis enzymes in the pancreas. (**A**) mRNA expression of *Glut2* (*n* = 6). (**B**) mRNA expression of the glycolytic enzymes glucokinase (*Gck*) and ketohexokinase (*Khk*). (**C**) mRNA expression of the amino acid transporter *SLC38A3*. (**D**) mRNA expression of the glutaminolysis enzymes glutaminase (*Gls*) and glutamate dehydrogenase 1 (*Glud1*). All data are expressed as mean ± SEM. *n* = 6 * *p* < 0.05 vs. control (Student’s *t*-test).

To identify the mechanism for the decrease in insulin secretion and the expression of *Pdx1*, we measured the mRNA expression of *Glut2* and the glycolytic enzymes glucokinase (*Gck*) and ketohexokinase (*Khk*) in the pancreas. Glut2 is involved in the translocation of glucose and fructose. *Gck* also plays an important role in regulating insulin secretion [23,24,25]. Khk is expressed in pancreatic islets and contributes to fructose metabolism [26,27]. Their expression in the HFCS group was significantly decreased compared with that in the control group (Figure 5A,B). Depending on the metabolic and nutritional status, amino acids, such as glutamine and glutamate, act as modulators of nutrient disposal, and play a role in regulating blood glucose through interaction with insulin signaling [28]. The mRNA expression of the amino acid transporter *SLC38A3,* and of the glutaminolysis enzymes glutaminase (*Gls*) and glutamate dehydrogenase 1 (*Glud1*), did not differ between the two groups (Figure 5C,D). The intake of excess HFCS–water under conditions of controlled caloric intake affected glucose and fructose metabolism, but not amino metabolism, in the pancreas.

### 3.7. Inflammatory Risk

**Figure 6:**
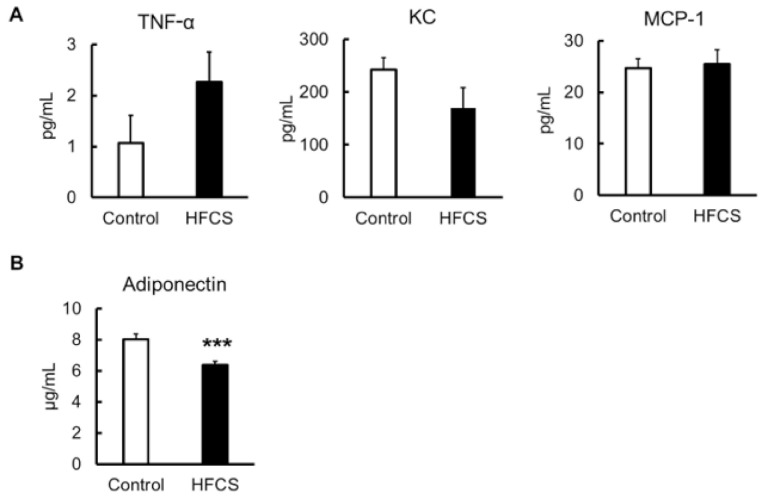
Serum inflammatory and anti-inflammatory cytokine parameters. (**A**) TNF-α, KC, and MCP-1. (**B**) Adiponectin. All data are expressed as mean ± SEM. *n* = 6. *** *p* < 0.005 vs. control (Student’s *t*-test).

To investigate the induction of inflammation, serum inflammatory cytokines, such as TNF-α, KC, and MCP-1, and the serum anti-inflammatory cytokine, adiponectin, were measured. The levels of inflammatory cytokines TNF-α, KC, and MCP-1 were not different between the two groups (Figure 6A), whereas the adiponectin level in the HFCS group was significantly decreased compared with that in the control (Figure 6B). Although the adiponectin level was decreased, the intake of excess HFCS–water for only 8 weeks did not cause any apparent acute or chronic inflammation.

## 4. Discussion

There has been a considerable discussion about whether HFCS is a risk factor for T2D and obesity. It has been suggested that sugar-containing beverages, including fruit juices, may induce T2D [12,14], and countries that use HFCS in their food items have a significantly higher prevalence of T2D than countries that do not use this sweetener. In addition, it has been reported that excessive intake of HFCS does not lead to obesity, but impairs glucose tolerance. On the other contrary, there are reports that no correlation exists between the intake of sugar, such as sucrose and HFCS, and the development of diabetes [19,29]. HFCS has been suggested to induce obesity, and it has been reported that excessive intake of 25% HFCS–water increases body weight and fat mass and causes glucose intolerance in mouse experiments [30]. However, there are also reports that drinking sweetened beverages does not lead to obesity [31,32]. Thus, it is unclear whether HFCS consumption leads to obesity or diabetes. However, in many of these studies, the caloric intake and balance of nutrient intake were not considered.

In this study, we investigated the effect of excessive consumption of HFCS–water on glucose tolerance and obesity under conditions of controlled caloric intake. This experimental condition envisaged the common dietary habit of drinking a large amount of HFCS-containing beverages and not taking in enough proper nutrition. Excessive HFCS–water intake under these conditions can lead to diabetes without obesity or dyslipidemia. The results of the present study, which was conducted under conditions of same caloric intake, are novel. Thus, it is presumed that HFCS does not cause obesity unless there is an overintake of calories. We suggest that obesity is not caused by HFCS, but by a combination of other conditions, such as excessive caloric intake and a high-fat diet. We speculate that the aforementioned inconsistencies, which are often found in epidemiological studies investigating the relationship between sugars and chronic diseases, including T2D, may partly be due to the ambiguity of not only the definition and type of sugar, but also the nutritional balance of diet and total daily caloric intake. However, the effects of long-term excessive HFCS–water intake should be investigated because, in this study, we only investigated the effects of short-term intake for 8 weeks.

Fructose intake has also been linked to the development of metabolic syndromes, including diabetes, obesity, and fatty liver disease [33]. Moreover, fructose consumption in a high-fat diet accelerates obesity and IR [34]. Fructose is taken up from the lumen of the small intestine by Glut5 [35]. The liver is considered to be the main organ of dietary fructose metabolism [36]; however, in the case of low dietary fructose, it is converted into glucose and organic acids in the small intestine and is nearly completely metabolized. In the case of a large amount of fructose that cannot be fully digested in the small intestine, it is transported to the liver for digestion [37]. The liver expresses the highest levels of Khk-C, and high fructose intake causes liver-related medical conditions [38,39]. The hepatic metabolism of fructose, which is not subject to negative feedback inhibition, is independent of the energy status, resulting in unregulated hepatic fructose uptake and increased lipogenesis [40,41]. In OFTT, that is, oral administration of fructose, blood glucose levels increased in both the HFCS and control groups, suggesting that fructose was converted to glucose in the small intestine. In the HFCS group, the blood glucose level increased 15 to 30 min after fructose administration compared to that in the control group (Figure 3E), but the insulin-secretory capacity in the HFCS group did not change before or after fructose administration (Figure 3F). In addition to a decrease in insulin-secretion capacity in response to glucose stimuli, this may be because of the time taken for the conversion of fructose into glucose in the small intestine. However, the relationship between fructose and insulin secretion needs to be investigated further.

The excessive HFCS–water intake resulted in impaired glucose tolerance due to insulin-secretion defect in OGTT; however, there was no change in the insulin-positive area or *Ins1* and *Ins2* expression in the pancreas. However, because the expression of *pdx-1*, *amy2a5*, and *amy2b* was decreased compared to that in the control group, HFCS clearly had some effect on the decrease in insulin secretion. *Pdx1* is a transcription factor required for the enhancement of insulin secretion and replication of β-cells [42,43]. Deletion of *Pdx1* in β-cells induces insulin-secretion disorders and diabetes [44,45]. Serum pancreatic amylase levels are known to be decreased in patients with diabetes [21,22]. Pancreatic amylase activity is reduced in a streptozocin-induced diabetic rat model [46]. Moreover, in the streptozocin-induced diabetic pig model, the activities of amylase and chymotrypsin from the pancreas are decreased, while lipase activity is enhanced [47]. In pancreatic diabetes, secretion of amylase and lipase is reduced due to inflammation. In some cases, diabetes is caused by damage to exocrine cells, as well as endocrine cells. We also examined the mRNA expression of *Prss2* and *Pnlip* in the pancreas, finding that the expression was not different between the two groups (Figure 4E). In addition to an unbalanced diet, excessive intake of HFCS–water had negative effects on both endocrine and exocrine cells. However, why it leads to a decrease in the expression of carbohydrate-related enzymes needs to be further investigated.

GK rats, which are a well-known model for nonobese T2D, have impaired insulin secretion because of reduced ATP production due to metabolic abnormalities of glucose [48]. The mechanism of reduction of insulin secretion in mice with nonobese T2D might be similar to that in GK rats. Thus, we investigated the effect of HFCS drink on glucose metabolism in the pancreas. β-cells do not express Glut5, a fructose transporter; however, fructose is transported by Glut2 [49,50], and β-cells can metabolize fructose, albeit much less efficiently than glucose [26]. Glut2 transports glucose and fructose into β-cells and is important for insulin secretion [51,52]. The expression of Glut2 is significantly reduced in β-cells in mice with diabetes induced by a high-fat diet [53]. Glucose and fructose transported into the cells are metabolized by Gck and Khk [54]. ATP is then produced, the ATP-sensitive potassium channel is closed, and membrane depolarization ensues. Thereafter, the voltage-gated calcium channel opens and calcium flows into the cell, resulting in the release of insulin into the blood [55]. In general, when blood glucose level is low, glucokinase activity is decreased and insulin secretion is suppressed, while when blood glucose level is high, glucokinase activity is promoted and insulin secretion is accelerated. However, in case hyperglycemia continues for a long period of time, the function of β-cells decreases due to glucose toxicity involving reactive oxygen species (ROS) [56,57]. The expression of *Glut2*, *Gck*, and *Khk* mRNAs was decreased in the HFCS group (Figure 5A,B), and it was speculated that excessive HFCS–water intake might cause insulin-secretion defect in response to glucose stimuli due to decreased expression of the glucose transporter and their metabolic enzymes in glucose metabolism. Moreover, because excessive HFCS–water intake affected not only endocrine cells but also exocrine cells, resulting in decreased expression of amylase, we surmise that it may be due to something other than ROS.

Both glutamate and glutamine provide reducing equivalents for the production of NADH in the TCA cycle and ultimately for the synthesis of ATP to facilitate glucose-stimulated insulin secretion via the closure of ATP-sensitive potassium channels [28,58]. Glutamine is transported into β-cells by transporters, such as SLC38A3 [28,59,60]. Glutamine is converted to glutamate by Gls and then to α-ketoglutarate by Glud. Glutamine deprivation induces a reduction in mitochondrial respiration and an increase in glucose uptake and utilization, and impairs β-cell function by reducing the production and secretion of insulin. In the HFCS group, the levels of TP and BUN in the blood were lower than those in the control (Table 2) because of the nutritional imbalance with low intake of protein and fat (Figure 1D). Therefore, in addition to the effects on glycolysis, we also investigated whether glutamine metabolism was affected by determining the mRNA expression of *SLC38A3*, *Gls*, and *Glud1*. Overall, excessive HFCS–water intake under conditions of controlled caloric intake had no effect on glutamine and glutamate metabolism.

A chronic inflammatory state is related to obesity and obesity-associated T2D, and elevated proinflammatory cytokine levels found in obesity-associated T2D are related mainly to obesity rather than to T2D [61]. Obesity induces chronic inflammation, which in turn induces IR [62]. On the contrary, adiponectin has important metabolic and anti-inflammatory actions that may have a protective role in the development of diabetes [63]. Higher adiponectin levels are associated with a lower incidence of diabetes [64,65]. In the HFCS–water group, although the blood adiponectin level was low (Figure 6B), there was no increase in the levels of inflammatory cytokines (Figure 6A), and no evidence of inflammation was observed upon histological examination. Because mice in the HFCS group were not obese, it is possible that inflammation was not induced. However, the effects of long-term HFCS intake on inflammation need to be investigated.

The present study assumes importance in view of the increasing prevalence of T2D, especially in the Asian population, and the fact that T2D is associated with several other complications that decrease the quality of life and increase the mortality rate. Nonetheless, these results must be interpreted with caution and a number of limitations should be borne in mind. It will be necessary to compare not only the effects of long-term excess HFCS intake, but also the effects of other sweeteners, such as sucrose, and of conditions that do not restrict energy intake, in future research. Recently, it has become clear that the microbiome influences inflammation, obesity, metabolic disease, and metabolic functions [66,67]. Excess HFCS intake during a short term of adolescence in mice induces fatty liver, alters metabolic pathways, many of which are also altered in adulthood, and changes the microbiome [68]. In addition, a fructose-rich diet during a short term of adolescence in rat prolongs fructose-induced dysregulation of hepatic metabolism [69]. In this study, adolescent male mice were used. It has been reported that metabolic capacity changes with sex differences [70]. Further studies are needed to clarify the effects of sex and age differences on excessive HFCS-intake-induced metabolic changes, as well as the underlying mechanisms.

## 5. Conclusions

Under controlled caloric intake and nutritional imbalance, the excessive intake of HFCS–water did not induce obesity, but did induce IGT. This condition was induced by insulin-secretion defect and decreased expression of *Glut2, Gck, and Khk* in the pancreas. However, no inflammation was observed. These results suggest that excessive consumption of HFCS drinks, such as soft drinks, without a proper diet, induces nonobese IGT due to insulin-secretion defect.

## Figures and Tables

**Table 1:** Primers used for real-time PCR.

Gene	Forward	Reverse
*Ins1*	TCAGAGACCATCAGCAAGCA	AGAGAGCCTCTACCAGGTGG
*Ins2*	CACCCAGGCTTTTGTCAAGC	TGCCAAGGTCTGAAGGTCAC
*Pdx1*	AAATCCACCAAAGCTCACGC	GCAGTACGGGTCCTCTTGTT
*Amy2a5*	CAAAATGGTTCTCCCAAGGA	CATCTTCTCGCCATTCCACT
*Amy2b*	GATGCTTATCAGGTTATTGATCTGG	TCTCTCCATTCCACTTGCGG
*Prss2*	TGATCTGTGTTGGCTTCCTG	CCAGTCCACGTAGTTGCAGA
*Pnlip*	TTCCCGAACGACATATACCC	GCTCCAAAGGTCCTCTTTCC
*Glut2*	GACTGGAGCCCTCTTGATGG	GTGTGGTTGGAGCGATCTCT
*Gck*	CAGGACAGTGGAGCGTGAA	TCCAGGAAGTCAGAGATGCAC
*Khk*	GCGTGGATGTGTCTCAAGTG	GCAGGTTCGTGTCGTAGAGT
*SLC38A3*	CGACAGACAGAGATGGTGGA	CCTCGAAATCGGTGAAGTGT
*Gls*	GCGGGCGACAATAAAATAAA	CACTCTTTCAACCTGGGATCA
*Glud1*	TGGCCTACACAATGGAGAGA	TCAGGTCCAATCCCAGGTTA
*18S rRNA*	AACGAACGAGACTCTGGCAT	CGGACATCTAAGGGCATCACAG

**Table 2:** Serum biochemical parameters.

Parameters	Control Group (*n* = 6)	HFCS Group (*n* = 6)
TG (mg/dL)	39.72 ± 6.81	45.58 ± 6.80
T-Cho (mg/dL)	84.18 ± 3.66	94.04 ± 2.77
UA (mg/dL)	1.24 ± 0.24	1.04 ± 0.14
BUN (mg/dL)	25.05 ± 1.44	20.49 ± 1.43 *
AST (U/L)	112.46 ± 16.50	76.74 ± 9.28
ALT (U/L)	39.2 ± 3.51	29.58 ± 1.46 **
TP (g/L)	4.67 ± 0.09	4.38 ± 0.06 *

** *p* < 0.01, * *p* < 0.05 vs. control (Welch’s *t*-test).

## Data Availability

The data presented in this study are available on request from the corresponding author.
